# Evaluating Training Need for Epidemic Control in Three Metropolitans: Implications for COVID-19 Preparedness in Vietnam

**DOI:** 10.3389/fpubh.2020.589331

**Published:** 2020-11-05

**Authors:** Diep Ngoc Nguyen, Huong Thi Le, Phong Khanh Thai, Xuan Thi Thanh Le, Men Thi Hoang, Linh Gia Vu, Toan Thi Thanh Do, Khanh Nam Do, Giap Van Vu, Tu Huu Nguyen, Thanh Tuan Le, Trung Dinh Tran, Dat Van Truong, Cuong Duy Do, Thu Ha Nguyen, Dung Tri Phung, Son Hong Nghiem, Thuc Thi Minh Vu, Bach Xuan Tran, Carl A. Latkin, Roger C. M. Ho, Cyrus S. H. Ho

**Affiliations:** ^1^Institute for Global Health Innovations, Duy Tan University, Da Nang, Vietnam; ^2^Faculty of Pharmacy, Duy Tan University, Da Nang, Vietnam; ^3^Institute for Preventive Medicine and Public Health, Hanoi Medical University, Hanoi, Vietnam; ^4^Queensland Alliance for Environmental Health Sciences (QAEHS), The University of Queensland, Brisbane, QLD, Australia; ^5^Center of Excellence in Evidence-Based Medicine, Nguyen Tat Thanh University, Ho Chi Minh City, Vietnam; ^6^Department of Internal Medicine, Hanoi Medical University, Hanoi, Vietnam; ^7^Respiratory Center, Bach Mai Hospital, Hanoi, Vietnam; ^8^Vietnam Young Physicians' Association, Hanoi, Vietnam; ^9^Vietnam National Heart Institute, Bach Mai Hospital, Hanoi, Vietnam; ^10^Faculty of Public Health, Danang University of Medical Technology and Pharmacy, Da Nang, Vietnam; ^11^Faculty of Pharmacy, University of Medicine and Pharmacy at Ho Chi Minh City, Ho Chi Minh City, Vietnam; ^12^National Hospital of Tropical Diseases, Bach Mai Hospital, Hanoi, Vietnam; ^13^School of Medicine, Griffith University, Gold Coast Campus, Southport, QLD, Australia; ^14^Centre for Applied Health Economics (CAHE), Griffith University, Brisbane, QLD, Australia; ^15^Bloomberg School of Public Health, Johns Hopkins University, Baltimore, MD, United States; ^16^Department of Psychological Medicine, Yong Loo Lin School of Medicine, National University of Singapore, Singapore, Singapore; ^17^Institute for Health Innovation and Technology (iHealthtech), National University of Singapore, Singapore, Singapore; ^18^Department of Psychological Medicine, National University Hospital, Singapore, Singapore

**Keywords:** COVID-19, infection, medical students, epidemic control, training need

## Abstract

Upon the outbreak of the COVID-19 pandemic, countries worldwide face a critical shortage of human resources in the health sector. Medical students are a potential task force with the capability to support the stretched health sector. This study aims to evaluate their training need for epidemic control in order to employ them effectively. A cross-sectional study was conducted using a web-based survey from December 2019 to February 2020. There were 5,786 observations collected using the snowball sampling technique. Logistic regression was applied to identify factors associated with training participation in epidemic prevention and disaster prevention. Multiple Poisson regression model was constructed to examine factors associated with the number of times they participated in sanitation training and disaster prevention activities in the previous 12 months. Sanitation and health education communication activities had the highest proportion of participants, with 76.5 and 38.4%, followed by examining and treating diseases in the community (13.4%). Those who participated in community activities had a higher number of times to participate in epidemic sanitation training and be involved in disaster prevention. This study informed the need for training programs to prepare medical students for COVID-19 epidemic responses. The training curriculum should include both theoretical approaches and contextual approaches to achieve efficient epidemic control.

## Introduction

COVID-19 is the infectious disease caused by a new coronavirus that was first reported from Wuhan, China, on 31 December 2019. The World Health Organization (WHO) has officially declared the outbreak of COVID-19 pandemic on 11 March 2020, after the disease caused by the new coronavirus spread to more than 100 countries and led to tens of thousands of cases within a few months. With more than 200 countries, areas, or territories with positive cases of COVID-19, social distancing has been applied in most countries to minimize the spread of the virus. Washing of hands frequently, routine cleaning of frequently touched surfaces, and, more recently, wearing of face mask/covering are the additional methods recommended by health authorities to slow down the spread of the virus. Recent COVID-19 research found that these precautional measures safeguard well-being at the COVID-19 outbreak (1) and peak of epidemics (2). Because COVID-19 is a completely new epidemic, governments in different countries have taken different approaches to control this epidemic in their countries. The UK government, for instance, had briefly considered the “herd immunity” approach but quickly had to admit that, with COVID-19, the health impact, including the mortality rate, would be phenomenal (3, 4). Other countries such as Taiwan, Singapore, and Vietnam chose to apply strict measures at the early stages of the outbreak rate such as forced isolation, social distancing, and strict quarantine and contact tracing rules. Those strict measures help those countries to keep the numbers of COVID-19 cases low but require extra human resources, especially well-trained preventive medicine staff that could lead to a shortage in many countries during this epidemic period.

Vietnam belongs to the low- and middle-income country (LMIC) group whose water, safety, and hygiene in healthcare facilities need to be improved. According to State Party Self-Assessment Annual Reporting 2018, the average scores of indicator groups, including points of entry, national health emergency framework, and surveillance, were quite low in Vietnam (40/100, 47/100, and 50/100, respectively) (5, 6). Vietnam achieved a medium score in human resources indicators (60/100), indicating a great potential in its human resources. As mentioned above, Vietnam was considered among countries that performed well in preventing the spread of coronavirus in the community. The government has immediately applied timely policies as well as informed the residents about the perception of using masks and other risks with the considerable contribution of social media and science journalism (7–11). Notwithstanding these timely actions, the government has mobilized the entire health sector, including medical students, to participate in epidemic control, and the Ministry of Health of Vietnam has called on medical students to help in the fight against the epidemic to enhance human resources (12–14).

In previous epidemics such as SARS, H5N1, and AIDS, the great contribution of medical students in preventing and controlling the disease has been recognized in Canada, Hong Kong, and Turkey. Medical students are important human resources that might have the capability to support the community, with their advantages of youth, skills, and knowledge, in the fight against an epidemic (15–17). Despite their confidence and willingness to contribute, an important lesson is to ensure preparedness and awareness among medical students who are directly involved in epidemic control because of their specific roles. In terms of COVID-19 epidemic, the process of medical student education is reconsidered to evaluate whether these students have the required preparedness to participate in the fight against the deadly coronavirus (18, 19).

The spread of COVID-19 around the world has shown that metropolitans have the highest risk of overwhelming spreading due to high population density and social activities. Fortunately, the metropolitans in Vietnam are also where most of the medical students are. In the three high-risk metropolitans of Vietnam, i.e., Hanoi, Da Nang, and Ho Chi Minh City (the three largest cities), most medical students have participated in a training of epidemic prevention and support, but there has been a limited assessment of the training process and the appropriateness of the training program. Therefore, during the unanticipated epidemic of COVID-19, this study aims to figure out whether medical students have received appropriate training for epidemic control and the associated factors that affected their training and practices.

## Materials and Methods

### Study Design and Sampling Methods

A cross-sectional study was conducted using a web-based survey named SurveyMonkey®. SurveyMonkey is one of most common platforms for online survey because it is intuitive and easy to share. The system provides the ability to develop questionnaires with a variety of question types and is easy for researchers to modify. At the same time, the confidentiality of the collected information is also guaranteed by passwords and a privacy system. Using this platform, research was ably conducted on a large scale in Vietnam in a short time. The reason for time limitation was because this study was a sub-analysis of a project on medical workers and medical students during the initial period of the COVID-19 epidemic when the assessment of national responses was vital. Data were collected from December 2019 to February 2020 in Vietnam, which covered the period from the very first COVID-19 cluster that was reported in Wuhan, China, on 31 December 2019 until the outbreak was declared a public health emergency of international concern by WHO on 30 January 2020 but before WHO declared the outbreak as an epidemic on 11 March 2020.

The participants were medical students in different medical universities in Vietnam who met the following inclusion criteria: (1) at least 18 years old, (2) currently living in Vietnam, (3) agreeing to participate in the survey by providing an online informed consent, and (4) having the ability to read and respond to the questionnaire.

In order to avoid having participants answer the survey more than once, no material incentives were given to the participants for their engagement of the survey. In total, there were 5,786 observations that can be used for analysis.

We applied the “snowball sampling technique” to recruit participants. Several core groups of medical students from the Vietnam Young Physician Association, Vietnam Youth Federation, and medical universities in Hanoi, Danang, and Ho Chi Minh City have been centered upon at the beginning of the recruitment process. The core groups sent the link to students of medical universities in other provinces in three regions of North, Central, and South Vietnam for them to access the questionnaire. The students who had been involved in the study were instructed to invite other students to join because they were more likely to know other people who have similar background characteristics and are suitable to be involved in the survey. One person could easily send the link of survey *via* weblink, email, social network, and messenger apps to others.

### Variables

#### Socioeconomic Characteristics

The participants provided information about their gender, age, specialty, marital status, and living areas.

#### Training and Practice for COVID-19 Epidemic Control Among Medical Students

The participants were asked if they attended training classes in epidemic prevention and disaster prevention and if they have ever been involved in disaster prevention. They self-reported community activities that they had participated in. They were also asked about the number of times that they participated in environmental sanitation training in the previous 12 months as well as were involved in disaster prevention. Moreover, the participants were asked whether they agree with the importance of the following local hygiene and disease prevention measures:

- Early prevention, environmental sanitation, and population health improvement
- Mobilization of community participation in disease control
- Training on up-to-date scientific knowledge
- Raising awareness of the impacts of climate change
- Ensuring adequate budget for disease prevention
- Periodic surveillance for infectious diseases
- Strengthening health communication and education programs
- Development of epidemic forecast systems to provide early warning
- Improvement of interdisciplinary scientific research capacity
- Workforce support for preventive medicine sectors
- Development of guidelines for disease prevention
- Increasing coordination among local actors.

### Statistical Methods

STATA software was used to analyze the final data. Mean and standard Deviation (SD) were described for quantitative variables; frequency and percentage were used to describe qualitative variables. The differences between these variables were tested using Kruskal–Wallis test and chi-square test, depending on each variable. *P* < 0.05 was considered statistically significant. Logistic regression was applied to identify factors associated with training participation in epidemic prevention and disaster prevention. A multiple Poisson regression model was constructed to examine the factors associated with the number of times that they participated in sanitation training and disaster prevention activities in the previous 12 months. The independent variables included in the model were socioeconomic status and the specialty of the participants. Forward stepwise selection was used to construct the reduced model that only contained independent variables having a log-likelihood ratio test *p* < 0.2.

### Ethical Consideration

The Scientific Council of Vietnam Central Youth Union has reviewed and approved this study protocol (No. 85 QĐ/TWĐTN-VNCTN).

## Results

Table 1 shows that there were 74.9% female medical students, about three times higher than the 25 male medical students (25.1%). The average age of the medical students was 20.6 years (SD = 1.7), and 98.5% among them were single. The rate of living in urban and rural areas was 87.7 and 12.3%, respectively.

**Table 1:** Socioeconomic characteristics of medical students.

	**Medical specialist**		**General doctor**		**Pharmacist**		**Others**		**Total**		***p*-value**
	***n***	**%**	***n***	**%**	***n***	**%**	***n***	**%**	***n***	**%**	
Total	2,019	34.9	1,189	20.6	1,198	20.7	1,380	23.9	5,786	100.0	
**Gender**											
Male	589	29.2	338	28.4	320	26.7	205	14.9	1,452	25.1	<0.01^a^
Female	1,429	70.8	851	71.6	878	73.3	1,175	85.1	4,333	74.9	
**Living area**											
Urban	1,782	88.8	1,039	88.1	1,056	88.6	1,161	85.1	5,038	87.7	0.01^a^
Rural	225	11.2	141	12.0	136	11.4	204	15.0	706	12.3	
**Marital status**											
Single	1,985	98.4	1,173	98.8	1,178	98.7	1,355	98.2	5,691	98.5	0.57^a^
Others	32	1.6	14	1.2	16	1.3	25	1.8	87	1.5	
**Region**											
Northern	276	14.1	495	42.9	171	14.6	537	39.9	1,479	26.2	<0.01^a^
Central	117	6.0	58	5.0	82	7.0	45	3.3	302	5.4	
South	1,570	80.0	601	52.1	920	78.4	765	56.8	3,856	68.4	
**Participated in community activities**											
Yes	885	43.9	525	44.2	455	38.1	555	40.3	2,420	41.9	<0.01^a^
No	1,131	56.1	663	55.8	739	61.9	823	59.7	3,356	58.1	
**Age group**											
Under 20	602	31.8	291	26.2	338	29.7	610	46.9	1,841	33.8	<0.01^a^
20 and above	1,290	68.2	821	73.8	802	70.4	692	53.2	3,605	66.2	
Age, mean (SD)	20.8	(1.9)	20.7	(1.5)	20.6	(1.6)	20.1	(1.8)	20.6	(1.7)	<0.01^b^

a *Chi-square test*.

b *Kruskal–Wallis test*.

Table 2 indicates that 87.6% of the medical students have attended training classes on hygiene in epidemic prevention and disaster prevention, and 91.6% of them were involved in disaster prevention with average number of times of 1.0 (SD = 1.4) and 1.1 (SD = 1.0) for each type of activity, respectively. Environment sanitation and health education communication are two learning topics that have the highest proportion of attendance among the participants at 76.5 and 38.4%, followed by examining and treating diseases in the community (13.4%). However, the remaining learning topics have relatively low rates of participants: mobilize community participation (11.5%), support for life and social security in the locality (7.4%), detect and notify epidemics/natural disasters (3.1%), and control and isolate affected areas (2.0%).

**Table 2:** Training and practice for epidemic control among medical students.

	**Medical specialist**		**General doctor**		**Pharmacist**		**Others**		**Total**		***p*-value**
	***n***	**%**	***n***	**%**	***n***	**%**	***n***	**%**	***n***	**%**	
Attend training classes on hygiene in epidemic prevention and disaster prevention	1,771	87.7	1,033	86.9	1,058	88.3	1,204	87.3	5,066	87.6	0.73^a^
Involved in disaster prevention	1,839	91.1	1,102	92.7	1,111	92.7	1,249	90.5	5,301	91.6	0.09^a^
**Activities of cleaning epidemic rooms in the community**											
Environmental sanitation	1,330	72.3	835	75.8	917	82.5	975	78.1	4,057	76.5	<0.01^a^
Health education communication	795	43.2	450	40.8	355	32.0	436	34.9	2,036	38.4	<0.01^a^
Examining and treating diseases in the community	310	16.9	135	12.3	122	11.0	141	11.3	708	13.4	<0.01^a^
Mobilize community participation	206	11.2	137	12.4	141	12.7	125	10.0	609	11.5	0.15^a^
Support for life and social security in the locality	149	8.1	76	6.9	103	9.3	66	5.3	394	7.4	<0.01^a^
Detect and notify epidemics/natural disasters	60	3.3	48	4.4	29	2.6	27	2.2	164	3.1	0.02^a^
Control and isolate affected areas	39	2.1	28	2.5	25	2.3	14	1.1	106	2.0	0.07^a^
Number of times participating in epidemic sanitation training (per year), mean (SD)	1.1	1.4	1.0	1.4	0.9	1.4	0.9	1.3	1.0	1.4	0.43^a^
Number of times involved in disaster prevention (per year), mean (SD)	1.3	1.0	1.2	1.0	0.9	1.0	0.9	0.8	1.1	1.0	0.01^b^

a *Chi-square test*.

b *Kruskal–Wallis test*.

Table 3 demonstrates the proportion of agreement on the importance of local hygiene and disease prevention measures among the participants. Early prevention, environmental sanitation, and population health improvement was the most common choice by medical students (36.5%), followed by mobilization of community participation in disease control (32.9%) and training on up-to-date scientific knowledge (32.5%).

**Table 3:** Agreement on the importance of local hygiene and disease prevention measures.

	**Medical specialist**		**General doctor**		**Pharmacist**		**Others**		**Total**		***p*-value**
	***n***	**%**	***n***	**%**	***n***	**%**	***n***	**%**	***n***	**%**	
Early prevention, environmental sanitation, and population health improvement	744	36.9	392	33.0	480	40.1	494	35.8	2,110	36.5	<0.01^a^
Mobilization of community participation in disease control	662	32.8	365	30.7	407	34.0	470	34.1	1,904	32.9	0.25^a^
Training on up to date scientific knowledge	708	35.1	318	26.8	410	34.2	443	32.1	1,879	32.5	<0.01^a^
Raising awareness on the impacts of climate change	635	31.5	317	26.7	390	32.6	416	30.1	1,758	30.4	0.01^a^
Ensuring adequate budget for disease prevention	595	29.5	288	24.2	387	32.3	400	29.0	1,670	28.9	<0.01^a^
Periodic surveillance for infectious diseases	605	30.0	297	25.0	355	29.6	411	29.8	1,668	28.8	0.01^a^
Strengthening health communication and education programs	571	28.3	283	23.8	330	27.6	395	28.6	1,579	27.3	0.02^a^
Development of epidemic forecasts systems to provide early warning	545	27.0	274	23.0	327	27.3	370	26.8	1,516	26.2	0.05^a^
Improvement of interdisciplinary scientific research capacity	550	27.2	250	21.0	323	27.0	349	25.3	1,472	25.4	<0.01^a^
Workforce support for preventive medicine sectors	545	27.0	243	20.4	311	26.0	357	25.9	1,456	25.2	<0.01^a^
Development of guidelines for disease prevention	519	25.7	246	20.7	303	25.3	344	24.9	1,412	24.4	0.01^a^
Increasing coordination among local actors	510	25.3	236	19.9	275	23.0	342	24.8	1,363	23.6	<0.01^a^

a *Kruskal–Wallis test*.

Table 4 presents the associated factors with training and practice for epidemic control. It can be seen that the medical students who had participated in community activities have a higher number of times to participate in epidemic sanitation training (Coef. = 0.22, 95% CI = 0.09; 0.35) and be involved in disaster prevention (Coef. = 0.21, 95% CI = 0.17; 0.24) compared with those who did not.

**Table 4:** Associated factors with training and practice for epidemic control among medical students.

	**Attend training** **classes on hygiene** **in epidemic prevention** **and disaster prevention^a^**		**Number of times** **participating in epidemic** **sanitation training (per year)^b^**		**Involved in disaster prevention^a^**		**Number of times** **involved in disaster** **prevention (per year)^b^**	
	**OR**	**95% CI**	**Coef**.	**95% CI**	**OR**	**95% CI**	**Coef**.	**95% CI**
Gender (female vs. male)			−0.11	−0.28, 0.06			−0.06^***^	−0.11, −0.02
Marital status (living with spouse vs. single)			0.28^*^	−0.03, 0.60	2.26	0.71, 7.23		
Age group (20 and above vs. under 20)			−0.13^**^	−0.26, −0.00	1.14	0.93, 1.40		
Participated in community activities (yes vs. no)	0.88	0.74, 1.03	0.22^***^	0.09, 0.35	1.46^***^	1.19, 1.79	0.21^***^	0.17, 0.24
**Region (vs. Northern)**								
Central	0.71^*^	0.49, 1.03						
South	0.83^*^	0.68, 1.01			1.19	0.96, 1.47		
**Specialty (vs. medical specialist)**								
General doctor					1.25^*^	0.96, 1.62		
Pharmacist			−0.20^**^	−0.37, −0.03	1.24	0.96, 1.60		
Others			−0.14^*^	−0.29, 0.00			−0.08^***^	−0.12, −0.04
**Agreement on the importance of local hygiene and disease prevention measures (yes vs. no)**								
Development of epidemic forecast systems to provide early warning							−0.04	−0.10, 0.02
Ensuring adequate budget for disease prevention							0.06^**^	0.01, 0.11
Workforce support for preventive medicine sectors							0.07^**^	0.01, 0.13
Increasing coordination among local actors					1.86^***^	1.42, 2.44		

*** *p < 0.01*,

** *p < 0.05*,

* *p < 0.1*.

a *Multivariate logistic regression*.

b *Multivariate Poisson regression*.

## Discussion

The findings of our study indicated that the medical students who had participated in community activities were likely to participate in training classes on environmental sanitation for epidemic prevention and be involved in disaster prevention; however, only nearly half of them did actually participate in these activities. Moreover, the rate of students involved in training is high, but the frequency of the training time was only about once per year, which is relatively low. Medical students need to be trained more in epidemic control in order to respond to all its aspects and enhance their supporting capability.

The majority of medical students were well-trained in environmental sanitation and health education communication and had basic knowledge about common diseases. The remaining practical activities (including mobilizing the community, supporting for life and social security in the locality, detecting, and notifying epidemics/natural disasters, and controlling and isolating affected areas) are also necessary for epidemic control. However, the rate of students' attendance in these training topics was low. It is suggested that the training program could be improved to help the students acquire all necessary knowledge to control the epidemic effectively.

Despite the fact that most medical students in Vietnam were not fully equipped with epidemic control training, during the COVID-19 epidemic, Vietnamese medical students have provided important assistance for the healthcare team in the following tasks: (1) assisting passengers in filling the health declaration forms (quarantine task), (2) assisting Center for Disease Control's (CDC) staff in tracing incidences of COVID-19 and people related to patients, (3) setting up genealogy maps to monitor the spread of the disease, (4) helping CDC's team to investigate the epidemic by tracking people who pose a high risk of transmission, (5) taking samples for testing for the virus, (6) quarantining suspected patients and assisting doctors and nurses, and (7) promoting preventive measures in the communities (20). Since the worldwide outbreak of COVID-19, in order to make these tasks more efficient, Vietnam has implemented timely interventions such as posting anti-epidemic guides through the webpage of medicine and pharmacy universities (including video clips, e-documents, updated news) as well as the mobile app NCOVI from the government portal that provided instructions for coronavirus disease prevention (21–25).

Even in more developed countries, the preparedness of medical students was also not high as they are perceived as non-frontline staff (26) and have lower resilience (27). A study in the United States reported that 98.5% of undergraduate students received quite poor health education communication (28). Findings from a study in England also pointed out the gap in public health and epidemiology training curriculum, and reformation to improve the training for medical students has been started (29). In the LMIC group, 86% of Pakistan medical students were not satisfied with the epidemiology approach at the university (30). In a Malaysian medical university, 84.6% of students believed that there should be more practical sessions (31). Medical universities in Vietnam previously assessed the importance of epidemiology and the training needs (32–35); however, the development in training programs has not been shown in our results yet.

The findings of this study help us draw up some potential implications. While this study indicated that the medical students in Vietnam had enough knowledge in environmental sanitation, health education communication, and common diseases through training in universities and community activities, it revealed, however, the necessity of training in other aspects including mobilizing the community, supporting for life and social security in the locality, detecting and notifying epidemics/natural disasters, and controlling and isolating affected areas. To deliver the knowledge of epidemic control to students, there are possible methods such as on-site training, e-learning, integrating with official training curriculums in the university, and developing instruction materials (36–41). Nevertheless, training programs should include theoretical approaches (e.g., pathogens, critical treatments) as well as other contextual approaches to achieve efficient epidemic control in each region. Training curriculum could be built based on the diversification of cultures to help the trainees be closer to the residents and meet expected outcomes in preventing and controlling the epidemic (42, 43), and after these classes, the trainees, including medical students, should have a higher level of awareness and preparedness on epidemic control. There was insufficient evidence about the customs and the colloquialisms of each population in epidemic control training programs. For example, current official online training documents of WHO have not identified customary risks yet (44). Therefore, medical staff at the grassroots level might meet difficulties in implementing solutions for public health approaches in epidemic response and system thinking in epidemic response. Further studies should assess customary characteristics in order to help medical staffs detect the risks at multilevel approaches.

Nonetheless, we acknowledge some limitations of our study. First, using the snowball sampling method might lessen the representability of our study. In return, our findings are urgent and consistent with the urgent requirements of the epidemic situation in Vietnam. Secondly, the self-reported data collection might lead to recall bias. A minor limitation in this study was the fact that the number of females was significantly higher than that of males. However, the results after dividing the variables by gender showed that there were very few variables which indicated a statistically significant difference between males and females. Finally, the cross-sectional study design might limit the possibility to identify causal relationships. Several questions remain unanswered at present about the training need among other population groups about epidemic control.

## Conclusions

Medical students are important human resources for the healthcare system in the period of health crisis. Indeed thousands of Vietnamese medical students have participated in the fight against COVID-19 epidemic. Although the country has been successful in controlling the virus, our study suggested that the participation of medical students could be more effective if there was more training about epidemic control among them to fill the gap in their training need. The results of our study provided evidence for reforming the training programs for epidemic control to prepare the medical students for COVID-19 epidemic responses in Vietnam. The training curriculum should include both theoretical approaches (e.g., pathology and critical treatments) as well as other contextual approaches to achieve efficient epidemic control in specific regions.

## Data Availability Statement

The raw data supporting the conclusions of this article will be made available by the authors, without undue reservation.

## Ethics Statement

The studies involving human participants were reviewed and approved by The Scientific Council of Vietnam Central Youth Union. The patients/participants provided their written informed consent to participate in this study.

## Author Contributions

BT, RH, and CH conceptualized this work. XL, GV, SN, and TV contributed to data curation. MH contributed to formal analysis. BT contributed to funding acquisition. DN, LV, TD, KD, TuN, DT, and CD contributed to the investigation. XL, DP, and SN contributed to the methodology. BT took charge of project administration and supervision. ThN contributed to resources. TL and TT took charge of software. CL, RH, and CH contributed to validation. DN contributed to writing the original draft. BT, PT, CL, RH, and CH reviewed and edited the manuscript. All authors contributed to the article and approved the submitted version.

## Conflict of Interest

The authors declare that the research was conducted in the absence of any commercial or financial relationships that could be construed as a potential conflict of interest.

## References

[1] Wang C Pan R Wan X Tan Y Xu L Ho CS. Immediate psychological responses and associated factors during the initial stage of the 2019 coronavirus disease (COVID-19) epidemic among the general population in China. Int J Environ Res Public Health. (2020) 17:1729. 10.3390/ijerph17051729 32155789 PMC7084952

[2] Wang C Pan R Wan X Tan Y Xu L Roger SM A longitudinal study on the mental health of general population during the COVID-19 epidemic in China. Brain Behav Immun. (2020) 87:40–8. 10.1016/j.bbi.2020.04.028 32298802 PMC7153528

[3] O'Grady C The U.K. Backed off on Herd Immunity. To Beat COVID-19, We'll Ultimately Need it. (2020). Available online at: https://www.nationalgeographic.com/science/2020/03/uk-backed-off-on-herd-immunity-to-beat-coronavirus-we-need-it/ (accessed March 20, 2020).

[4] Matthews O Britain Drops Its Go-It-Alone Approach to Coronavirus. (2020). Available online at: https://foreignpolicy.com/2020/03/17/britain-uk-coronavirus-response-johnson-drops-go-it-alone/ (accessed March 17, 2020).

[5] World Health Organization IHR State Party Self-Assessment Annual Reporting tool (SPAR) 2018. (2018). Available online at: https://extranet.who.int/sph/news/ihr-self-assessment-annual-reporting-tool-spar-2018 (accessed October 26, 2018).

[6] World Health Organization United Nations Children's Fund Water, Sanitation and Hygiene in Health Care Facilities: Status in Low and Middle Income Countries and Way Forward. Geneva: WHO (2015).

[7] Huynh TLD. “If You Wear a Mask, Then You Must Know How to Use It and Dispose of It Properly!”: a survey study in Vietnam. Rev Behav Econom. (2020) 7:145–58. 10.1561/105.0000012131752228

[8] Huynh TLD. The COVID-19 containment in Vietnam: what are we doing? J Glob Health. (2020) 10:010338. 10.7189/jogh.10.010338 32373318 PMC7182304

[9] La VP Pham TH Ho MT Nguyen MH P Nguyen KL Policy response, social media and science journalism for the sustainability of the public health system amid the COVID-19 outbreak: the vietnam lessons. Sustainability. (2020) 12:2931 10.3390/su12072931

[10] Huynh TLD. Does culture matter social distancing under the COVID-19 pandemic? Saf Sci. (2020) 130:104872. 10.1016/j.ssci.2020.104872 32550745 PMC7284251

[11] Huynh TLD The COVID-19 risk perception: a survey on socioeconomics and media attention. Econ Bull. (2020) 40:758–64.

[12] Vietnam News Medical Students Pitch in to Fight COVID-19. (2020). Available online at: https://vietnamnews.vn/society/654099/medical-students-pitch-in-to-fight-covid-19.html (accessed March 25, 2020).

[13] Tuoi Tre News Vietnam Calls Up Medical Students, Retired Doctors in Coronavirus Fight. (2020). Available online at: https://tuoitrenews.vn/news/society/20200322/vietnam-calls-up-medical-students-retired-doctors-in-coronavirus-fight/53614.html (accessed March 22, 2020).

[14] IDN Vietnam Insider 90,000 Vietnamese Doctors are Ready for Covid-19 Battle. Ho Chi Minh City: Vietnam Insider (2020).

[15] Mak KK Yiu YF Ko KL Hui KS Mak KM Mak LY. Attitudes and perceptions of influenza vaccination among Hong Kong doctors and medical students before the 2009 pandemic. Eur J Public Health. (2013) 23:257–62. 10.1093/eurpub/cks014 22383477

[16] Akan H Gurol Y Izbirak G Ozdatli S Yilmaz G Vitrinel A. Knowledge and attitudes of university students toward pandemic influenza: a cross-sectional study from Turkey. BMC Public Health. (2010) 10:413. 10.1186/1471-2458-10-413 20626872 PMC2918554

[17] Herman B Rosychuk RJ Bailey T Lake R Yonge O Marrie TJ. Medical students and pandemic influenza. Emerging Infect Dis. (2007) 13:1781–3. 10.3201/eid1311.070279 18217571 PMC3375803

[18] Miller DG Pierson L Doernberg S. The role of medical students during the COVID-19 pandemic. Am Coll Phys. (2020) 173:145–6. 10.7326/M20-1281 32259194 PMC7151405

[19] Rose S. Medical student education in the time of COVID-19. JAMA. (2020) 323:2131–2. 10.1001/jama.2020.5227 32232420

[20] Vietnamnews Youth Actively Support in Prevention of COVID-19 for the Community. (2020). Available online at: https://vietnamtimes.org.vn/youth-actively-support-in-prevention-of-covid-19-for-the-community-18812.html (accessed April 05, 2020).

[21] Danang People's Committee Instructions for Coronavirus Prevention. (2020). Available online at: https://www.danang.gov.vn/web/guest/video?_c=3andid=559 (accessed February 12, 2020).

[22] Ministry of Health Medical Declaration Through NCOVI Application. (2020). Available online at: https://vnpt.com.vn/tin-tuc/khai-bao-y-te-qua-ung-dung-ncovi-toan-dan-chung-tay-day-lui-dich-covid-19.html (accessed March 19, 2020).

[23] Hue University of Medicine and Pharmacy Prevention for COVID-19 Epidemic. (2020). Available online at: http://bvydhue.com.vn/c186/thong-tin-y-te-.html (accessed March 2020).

[24] Can Tho University of Medicine and Pharmacy (2020). Prevention for Coronavirus. Available online at: http://www.ctump.edu.vn/Default.aspx?tabid=2824 (accessed March 2020).

[25] Hai Phong University of Medicine and Pharmacy Ready Training to Respond to the Covid-19 for Medical Students in Hai Phong City. (2020). Avaiable online at: http://hpmu.edu.vn/hpmu/news/TIN-TUC-SU-KIEN/Tap-huan-san-sang-tiep-ung-dich-Covid-19-cho-sinh-vien-Y-khi-can-tiep-ung-tren-dia-ban-Thanh-pho-Hai-Phong-2567/ (accessed March 18, 2020).

[26] Tan BYQ Chew NWS Lee GKH Jing M Goh Y Yeo LLL. Psychological impact of the COVID-19 pandemic on health care workers in Singapore. Ann Intern Med. (2020) 173:317–20. 10.7326/M20-1083 32251513 PMC7143149

[27] Ho CS Chee CY Ho RC. Mental health strategies to combat the psychological impact of COVID-19 beyond paranoia and panic. Ann Acad Med Singap. (2020) 49:155–60. 32200399

[28] Ury WA Berkman CS Weber CM Pignotti MG Leipzig RM. Assessing medical students' training in end-of-life communication: a survey of interns at one urban teaching hospital. Acad Med. (2003) 78:530–7. 10.1097/00001888-200305000-00019 12742792

[29] Reis S. Curriculum reform: why? what? how? and how will we know it works? ISR J Health Policy Res. (2018) 7:30. 10.1186/s13584-018-0221-4 29880061 PMC5991462

[30] Butt AK Khan AA. Teaching biostatistics and epidemiology in a postgraduate medical institution: are we going in the right direction? East Mediterr Health J. (2008) 14:1192–7. 19161093

[31] Daher AM Amin F. Assessing the perceptions of a biostatistics and epidemiology module: views ofYear 2 medical students from a Malaysian university. a cross-sectional survey. BMC Med Educ. (2010) 10:34. 10.1186/1472-6920-10-34 20462464 PMC2885405

[32] Fan AP Tran DT Kosik RO Mandell GA Hsu HS Chen YS. Medical education in Vietnam. Med Teach. (2012) 34:103–7. 10.3109/0142159X.2011.613499 22288987

[33] Goto A Phuong NTN Huong NTM Hughes J. Building postgraduate capacity in medical and public health research in Vietnam: an in-service training model. J Public Health. (2005) 119:174–83. 10.1016/j.puhe.2004.05.005 15661126

[34] Dao AT Huong le T Nguyen HV Hoat LN. Epidemiology training needs assessment in Vietnam. Med Educ. (2010) 44:518–9. 10.1111/j.1365-2923.2010.03660.x 20519002

[35] Tran BX Dang AK Thai PK Le HT Le XTT Do TTT. Coverage of health information by different sources in communities: implication for COVID-19 epidemic response. J Int J Environ Res Public Health. (2020) 17:3577. 10.3390/ijerph17103577 32443712 PMC7277747

[36] Dodani S Songer T Ahmed Z Laporte RE. Building research capacity in developing countries: cost-effectiveness of an epidemiology course taught by traditional and video-teleconferencing methods in Pakistan. Telemed Health. (2012) 18:621–8. 10.1089/tmj.2011.0262 23061643

[37] Adam T Koopmanschap MA Evans DB. Cost-effectiveness analysis: can we reduce variability in costing methods? Int J Technol Assess Health Care. (2003) 19:407–20. 10.1017/S0266462303000369 12862197

[38] Armstrong T Bull F Development of the world health organization global physical activity questionnaire (GPAQ). J Public Health. (2006) 14:66–70. 10.1007/s10389-006-0024-x

[39] Rosswurm MA Larrabee JH Zhang J. Training family caregivers of dependent elderly adults through on-site and telecommunications programs. J Gerontol Nurs. (2002) 28:27–38. 10.3928/0098-9134-20020701-07 12168715

[40] Aggarwal R Gupte N Kass N Taylor H Ali J Bhan A. A Comparison of online versus on-site training in health research methodology: a randomized study. BMC Med Educ. (2011) 11:37. 10.1186/1472-6920-11-37 21682858 PMC3141795

[41] Pluye P Potvin L Denis J Making public health programs last: conceptualizing sustainability. Eval Program Plann. (2004) 27:121–33. 10.1016/j.evalprogplan.2004.01.001

[42] McKinn S Linh DT Foster K McCaffery K. Communication between health workers and ethnic minorities in Vietnam. Health Literacy Res Pract. (2017) 1:e163–72. 10.3928/24748307-20170629-01 31294262 PMC6607781

[43] Rheinländer T Samuelsen H Dalsgaard A Konradsen F. Hygiene and sanitation among ethnic minorities in Northern Vietnam: does government promotion match community priorities? Soc Sci Med. (2010) 71:994–1001. 10.1016/j.socscimed.2010.06.014 20619522

[44] World Health Organization Coronavirus Disease (COVID-19) Training: Online Training. (2020). Available online at: https://www.who.int/emergencies/diseases/novel-coronavirus-2019/training/online-training (accessed March 2020).

